# Case Report: Adrenal gland splenosis mimicking a neuroendocrine tumor on ^68^Ga-DOTATATE and ^18^F-FDG PET/CT imaging

**DOI:** 10.3389/fmed.2025.1578613

**Published:** 2025-05-09

**Authors:** Xianwen Hu, Wei Zhao, Pan Wang

**Affiliations:** ^1^Department of Nuclear Medicine, Affiliated Hospital of Zunyi Medical University, Zunyi, China; ^2^Department of Pathology, Affiliated Hospital of Zunyi Medical University, Zunyi, China

**Keywords:** splenosis, adrenal gland, neuroendocrine tumor, PET/CT, CT

## Abstract

Splenosis occurring in adrenal glands is relatively rare and is easily misdiagnosed as neoplastic lesions. Herein, we present a case of a 39-year-old woman who underwent a pancreatic tail resection and splenectomy 8 years ago due to caudal pancreatic neuroendocrine tumor and splenic invasion. She underwent abdominal ultrasound examination in an external hospital a month ago due to abdominal discomfort and found a lump in the left adrenal gland. She was admitted to our hospital for further diagnosis and treatment. Abdominal computed tomography (CT) examination revealed a nodule of equal soft tissue density on her left adrenal gland, which presented obvious uniform enhancement on contra-enhanced CT. Subsequently, she underwent fluorine-18 fluorodeoxyglucose (^18^F-FDG) and gallium-68 labeld 1, 4, 7, 10-tetraazacyclododecane-1, 4, 7, 10-tetraaceticacid -D-Phel-Tyr3-Thr8-OC (^68^Ga-DOTATATE) positron emission tomography (PET)/CT imagings, and showed slightly increased ^18^F-FDG uptake and obviously increased ^68^Ga-DOTATATE uptake in the lesion, suggesting the possibility of neuroendocrine tumor metastasis. However, postoperative pathology confirmed that the lesion was splenosis. Our case suggests that adrenal gland splenosis should be considered as a differential diagnosis of adrenal tumors, understanding the clinical and imaging features of splenosis can reduce misdiagnosis and avoid unnecessary surgical intervention.

## Introduction

Splenosis is an autologous implantation of spleen tissue caused by trauma or splenectomy. It is a completely isolated splenic tissue that exists outside the normal spleen and has no anatomical relationship such as blood supply and innervation with the normal spleen, and its blood supply comes from the small blood vessels that penetrate the capsule at the implantation site (1). There are three methods in which the spleen can be implanted into other organ tissues, including direct implantation of spleen tissue fragments separated after spleen injury, usually implanted into omentum, parietal peritoneum, intestinal wall serous layer and pelvic cavity, which are the main methods of spleen implantation; secondly, part of the debris of spleen tissue can be planted on the surface of organs far from the spleen area by washing the abdominal cavity with normal saline during the operation; and splenic myeloid cells can be disseminated through the splenic vein and are often implanted in the liver, while this type is rare (2). Splenosis can occur in any part of the abdominal cavity, mainly in the serous membrane of small intestine, omentum, parietal peritoneum, mesentery, pelvis, pancreas, stomach, etc., which is related to the normal anatomical location of the spleen and its implantation route, while it is rare in the adrenal gland or other distant diaphragmatic organs (3). Most splenosis have no clinical symptoms and are usually found by chance during physical examination. Depending on the location and size of the splenosis, some patients may develop gastrointestinal symptoms, such as abdominal pain, abdominal distension, intestinal obstruction, and anemia caused by gastrointestinal bleeding (4). Herein, we present a case of a 39-year-old woman who underwent a pancreatic tail resection and splenectomy 8 years ago due to caudal pancreatic neuroendocrine tumor and splenic invasion. A month ago, she underwent abdominal ultrasound examination due to abdominal discomfort and found a mass in the left adrenal gland, suspected to be a neuroendocrine tumor metastasis. To further evaluate the nature of the lesion, she underwent fluorine-18 fluorodeoxyglucose (^18^F-FDG) and gallium-68 labeld 1, 4, 7, 10-tetraazacyclododecane-1, 4, 7, 10-tetraaceticacid -D-Phel-Tyr3-Thr8-OC (^68^Ga-DOTATATE) positron emission tomography (PET)/computed tomography (CT) imagings, and showed slightly increased ^18^F-FDG uptake and obviously increased ^68^Ga-DOTATATE uptake in the lesion, suggesting the possibility of neuroendocrine tumor metastasis. However, postoperative pathology confirmed that the lesion was splenosis.

## Case report

**Figure 1:**
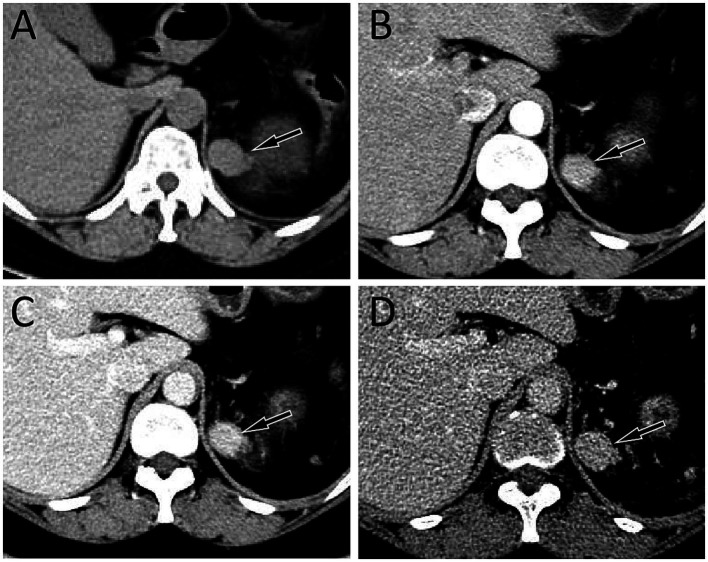
**(A)** Abdominal computed tomography (CT) revealed a soft tissue density nodule about 2.8 cm × 2.0 cm in size on the left adrenal gland (arrow); In the arterial phase **(B)**, venous phase **(C)**, and delayed phase **(D)** of contrast-enhanced CT, the lesion showed obvious and continuous uniform enhancement (arrows).

**Figure 2:**
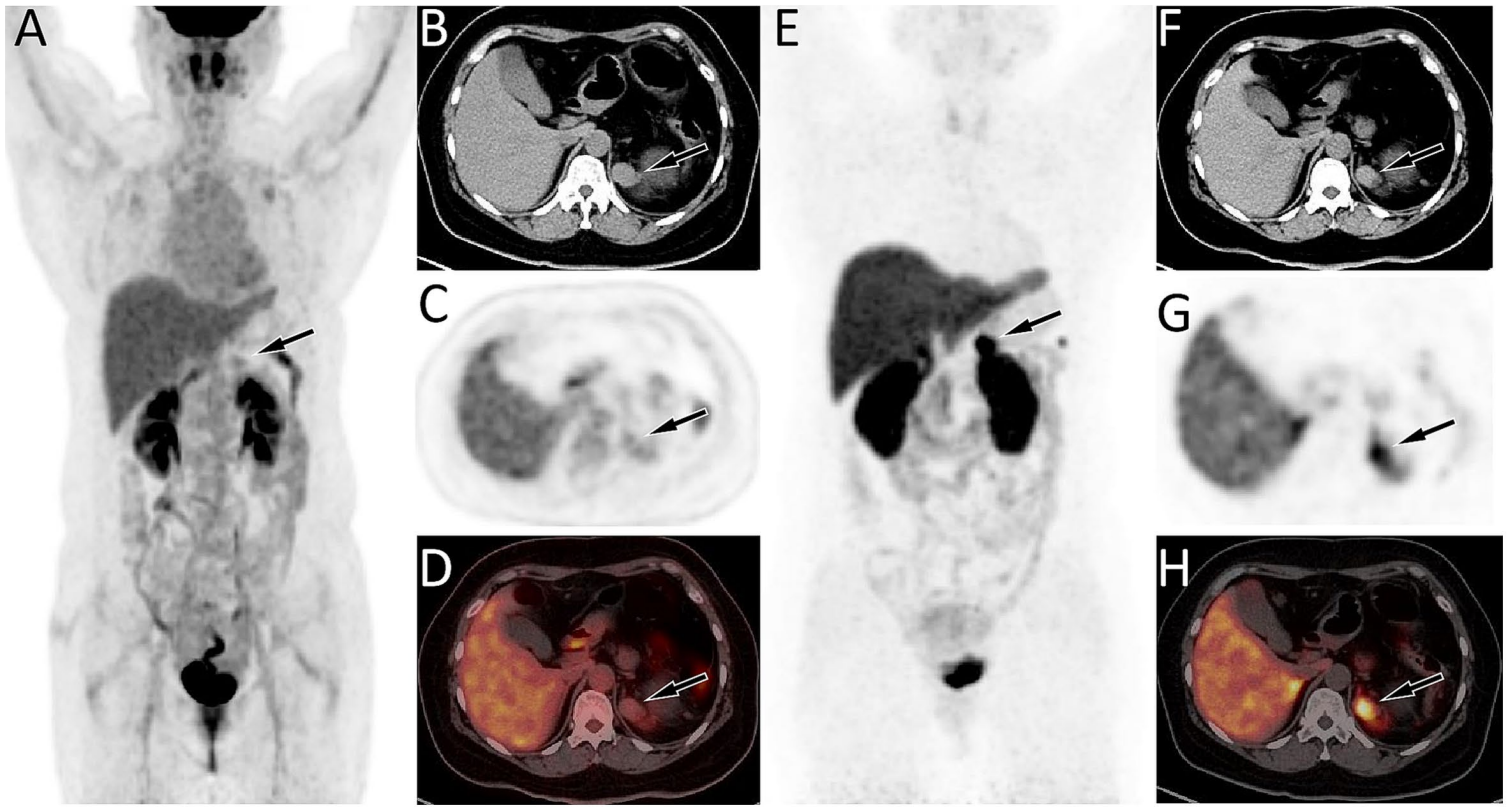
**(A–D)** Fluorine-18 fluorodeoxyglucose (^18^F-FDG) positron emission tomography (PET)/CT imaging of the patient; The maximum intensity projection (MIP, **A**) showed a slightly increased ^18^F-FDG uptake in the left upper abdomen (arrow). Axial CT **(B)** showed an isodense nodule in the left adrenal gland (arrow). The corresponding lesion had mildly increased ^18^F-FDG uptake on axial PET (**C**, arrow) and PET/CT fusion (**D**, arrow), with a maximum standardized uptake value (SUVmax) of 2.7. **(E–H)** ^68^Ga-DOTATATE PET/CT imaging; The MIP **(E)** showed a significantly increased ^68^Ga-DOTATATE uptake in the left upper abdomen (arrow). Axial CT **(F)**, PET **(G)** and PET/CT fusion **(H)** showed this increased focal uptake in the left adrenal gland (arrow), in the same location as the lesion shown on ^18^F-FDG PET/CT, with a SUVmax of 24.3.

**Figure 3:**
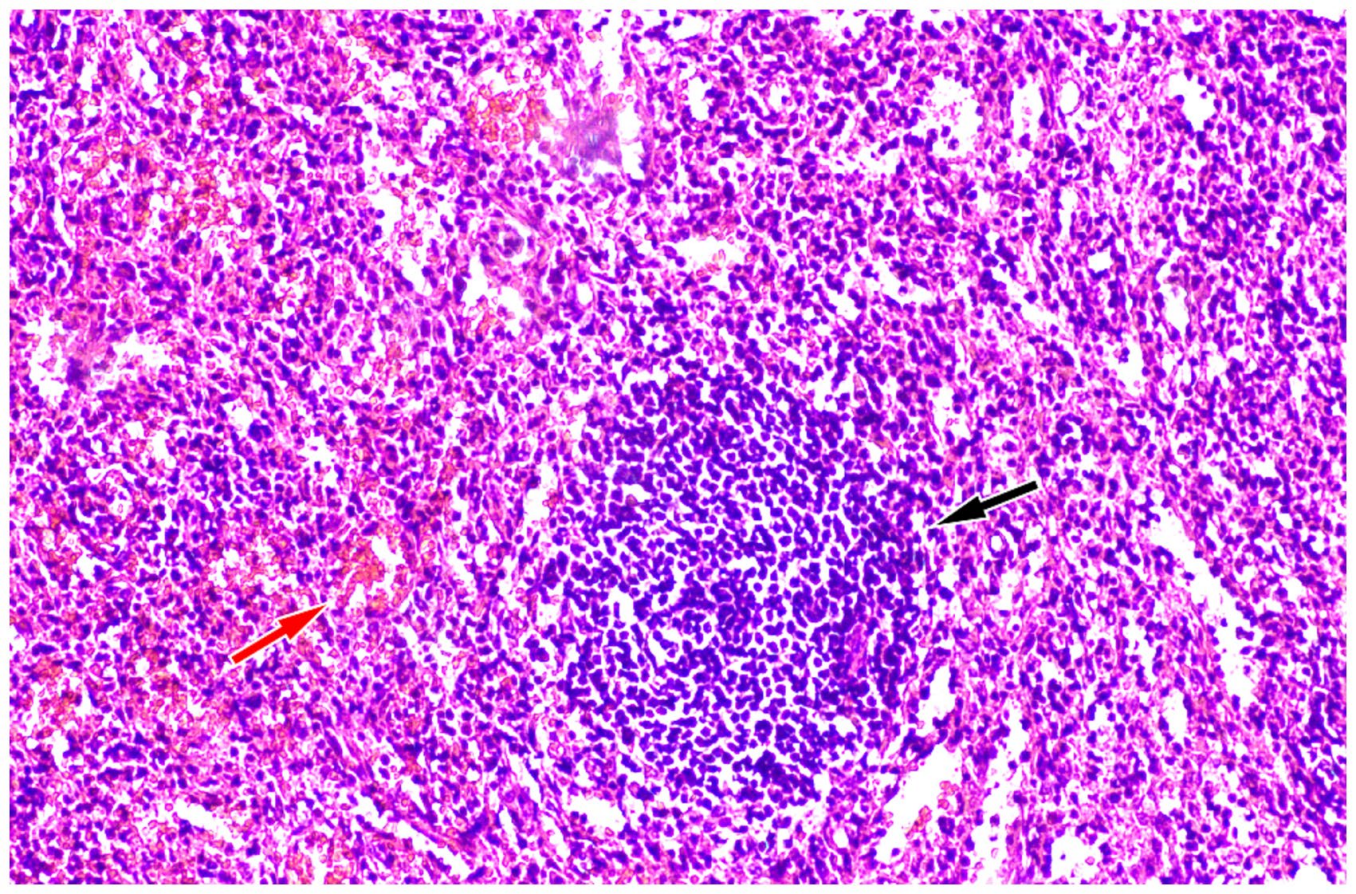
Hematoxylin–eosin staining (magnification, ×100) showing red pulp (black arrow) and white pulp (red arrow) within the tissue, suggesting splenic tissue.

A 39 year old woman was found to have a left adrenal gland mass during abdominal ultrasound examination at an external hospital 1 month ago due to abdominal discomfort. For further diagnosis and treatment, she visited the Department of Hepatobiliary and Pancreatic Surgery at our hospital on September 21, 2023. The patient had a medical history of pancreatic tail resection and splenectomy 8 years ago due to neuroendocrine tumor in the tail of the pancreas and invasion of the spleen, whose condition has remained stable since the surgery. Physical examination showed slight tenderness in the left upper abdomen, and no significant positive signs in the rest. On September 22, 2023, the patient’s fasting blood routine and digestive system tumor markers and other laboratory test results were all within the normal reference value range. On the same day, CT examination revealed a soft tissue density nodule about 2.8 cm × 2.0 cm in size in the patient’s left adrenal gland, which showed significant enhancement on contrast-enhanced CT scan (as shown in Figure 1). To further assess the nature of this lesion, the patient underwent a dual tracer (i.e., ^18^F-FDG and ^68^Ga-DOTATATE) PET/CT examination over the following 2 days, and the results showed slightly increased ^18^F-FDG uptake and obviously increased ^68^Ga-DOTATATE uptake in the lesion (Figure 2), which was suspected to be a neuroendocrine tumor metastasis. Because the lesion was localized, the patient underwent a left adrenalectomy on September 25, 2023. Postoperatively, the excised adrenal mass was sent for pathological examination. Microscopically, the resected tissue contained red pulp and white pulp (Figure 3), suggesting splenic tissue, which was consistent with the diagnosis of splenosis. Subsequently, the patient was discharged after receiving 3 days of anti-inflammatory treatment. Follow-up up to now, the patient has not complained of any discomfort.

## Discussion

The most common parenchymal organs for splenosis include pancreas, stomach, small intestine and kidney, but are relatively rare in adrenal glands (5). The possible reason for adrenal gland splenosis is that after splenectomy, the splenic myeloid cells were seeded to the adrenal gland by blood due to splenic vein embolism (1). Splenosis often presents as nodules with a diameter of about 1–3 cm, which are locally dark red or blue black, and can be single or multiple, which can occur within 5 months to 32 years after trauma or splenectomy, with an average of 10 years (6). Our patient was found to have adrenal gland splenosis 8 years after splenectomy, and the length of the nodule was about 2.8 cm, which was consistent with the characteristics of splenosis reported in the literature.

The imaging findings of ectopic splenosis in literature mostly focus on splenic implantation in the pancreas, stomach, intestines, liver, etc., the density of which on CT plain scan is similar to that of normal splenic tissue, exhibiting uniform or slightly higher soft tissue density (7–10). On contrast-enhanced CT scan, the enhancement mode of splenosis was related to the size of the lesion. Lesions smaller than 3 cm showed uniform enhancement in the arterial phase due to small red and white pulp content, short blood flow stroke and insignificant blood flow difference; However, lesions larger than 3 cm showed large red and white pulp content, long blood flow stroke and large difference in blood flow velocity, so presenting as uneven enhancement in the arterial phase. In the portal phase and the delayed phase, it showed continuous uniform or uneven strengthening (9). The MRI of splenosis was also similar to the signal of normal spleen tissue, that is, low signal on T1WI and high signal on T2WI. When the splenosis nodules contained hemosidin or mineral deposits, both T1WI and T2WI showed low signals, and when steatosis was present, the in-phase of T1WI showed high signals and the out-phase of T1WI signal decreased (4, 9, 11). The patient we reported presented with uniform soft tissue density nodules on CT plain and significant and sustained uniform enhancement on three-phase contrast-enhanced CT scan, consistent with literature reports.

Usually, when splenosis can be diagnosed through routine imaging examinations, further examination such as PET/CT is not necessary, and there are also rare descriptions of its PET/CT findings in the literature. However, our patient had a history of pancreatic neuroendocrine neoplasms, and as splenosis in the adrenal gland was rare, little was known about it, which led us to initially consider the possibility of metastatic tumor and eventually to perform PET/CT examination on the patient. Normal spleen tissue will have mild physiological uptake of ^18^F-FDG and significant uptake of ^68^Ga-DOTATATE. The mechanism of increased ^68^Ga-DOTATATE uptake in splenosis is related to the fact that somatostatin receptor type 2 (SSTR2) can be expressed in some immune cells such as macrophages and splenic sinus endothelial cells in normal spleen tissue, resulting in increased ^‌68^Ga-DOTATATE uptake. The function of splenosis is the same as that of normal spleen tissue, so its PET/CT findings are also similar, both showing increased ^68^Ga-DOTATATE uptake (12). As is well known, ^18^F-FDG and ^68^Ga-DOTATATE dual nuclear tracer PET/CT have been widely used in the diagnosis, staging, and post-treatment evaluation of neuroendocrine tumors, and G1-G2 grade neuroendocrine tumors can show mildly to moderately increased ^18^F-FDG uptake and significantly increased ^68^Ga-DOTATATE uptake (13–15). These imaging features are similar to the splenosis, combined with the patient’s history of neuroendocrine tumors, which ultimately led to our misdiagnosis of the disease.

The imaging findings of splenosis in the adrenal gland should be distinguished from the neoplastic lesions of the adrenal gland, mainly including neuroendocrine tumors such as pheochromocytoma, neuroblastoma, and ganglioneuroma, adrenal adenoma and metastatic tumor. Neuroendocrine tumors may present varying levels of increased ^18^F-FDG and ^68^Ga-DOTATATE uptake on PET/CT imaging (16). However, it varies in size and often shows bleeding, cystic changes, and necrosis within the tumor, resulting in uneven density on CT, and contrast enhanced CT scans usually show uneven enhancement (17, 18). For neuroblastoma, calcified foci are frequently observed within the tumor tissue (19).‌ Adrenal adenoma is usually a solitary nodule with small volume and uniform density, which appears as mild and uniform enhancement on contrast-enhanced CT scans (20). The incidence of adrenal metastasis in malignant tumors is 8.6–27.0%, when the lesion size is small, it shows uniform or slightly low density on CT. When the lesion size is large, it is easy to become cystic necrosis and show uneven density, and it can show different degrees of uniform enhancement or ring enhancement on contrast- enhanced CT (20). These variable CT signs make it difficult to distinguish it from the splenosis. For patients suspected of having ectopic splenosis nodules, preoperative technetium-99 m (^99m^Tc) labeled sulfur colloid (^99m^Tc-SC) scanning or ^99m^Tc labeled heat-damaged red blood cell (^99m^Tc-DRBC) imaging is feasible (21). There are a large number of macrophages in the spleen, which can phagocytose colloid and denatured red blood cells. ^99m^Tc-SC or ^99m^Tc-DRBC can be phagocytosed by mononuclear macrophages in the spleen after entering the body intravenously, and then remain in the spleen for imaging diagnosis (22).

Splenosis nodules have compensatory and proliferative functions, which plays a wide range of immune functions in hematopoiesis and red blood cell clearance, which can reduce the incidence of explosive infections (23, 24). Therefore, it is important to obtain an accurate diagnosis of splenosis before surgery, as most ectopic splenic implantation nodules can avoid surgical removal (25). Surgical intervention is required only if the implanted spleen compresses adjacent tissues causing symptoms such as pain, vomiting, etc. (26, 27). Like many cases reported in the literature, our patient also underwent unnecessary surgical resection treatment due to being misdiagnosed before surgery. As splenosis is a phenomenon of autoimplantation of splenic tissue fragments caused by splenic trauma or splenectomy, the prognosis of patients is good.

## Conclusion

Splenosis occurring in adrenal glands is relatively rare and is easily misdiagnosed as neoplastic lesions. Our case study suggests that adrenal gland splenosis should be considered as a differential diagnosis of adrenal tumors, especially in patients with a history of splenic trauma or splenectomy. Understanding the clinical and imaging features of splenosis can reduce misdiagnosis and avoid unnecessary surgical intervention.

## Data Availability

The original contributions presented in the study are included in the article/Supplementary material, further inquiries can be directed to the corresponding author.
