# Supplementary material for: Case Report: Adrenal gland splenosis mimicking a neuroendocrine tumor on 68Ga-DOTATATE and 18F-FDG PET/CT imaging
Source: Front Med (Lausanne). 2025 May 9;12:1578613. doi: 10.3389/fmed.2025.1578613 (PMC12098358; doi:10.3389/fmed.2025.1578613)

**
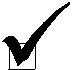

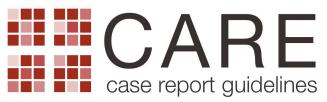
CARE Checklist of information to include when writing a case report**


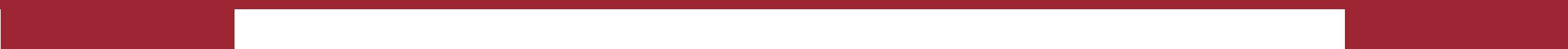

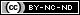


**Topic**

**Title**

**Key Words Abstract**

**(no references)**

**Introduction Patient Information**

**Clinical Findings**

**Timeline**

**Diagnostic**

**Assessment**

**Therapeutic**

**Intervention**

**Follow-up and Outcomes**

**Discussion**

**Patient Perspective Informed Consent**

| **Item** | **Checklist item description** |  |
| --- | --- | --- |
|  |  |  |
| **1** | The diagnosis or intervention of primary focus followed by the words “case report” . . . . . . . . . . . . . . . . . . . . . . . . | . . |
| **2** | 2 to 5 key words that identify diagnoses or interventions in this case report, including "case report" . | . . |
| **3a** | Introduction: What is unique about this case and what does it add to the scientific literature? . . . . . . . . . . . . . . | . . |
| **3b** | Main symptoms and/or important clinical findings . . . . . . . . . . . . . . . . . . . . . . . . . . . . . . . . . . . . . . . . . . . . . . . . . . . . . . . | |
| **3c** | The main diagnoses, therapeutic interventions, and outcomes . . . . . . . . . . . . . . . . . . . . . . . . . . . . . . . . . . . . . . . . . . . | |
| **3d** | Conclusion—What is the main “take-away” lesson(s) from this case? . . . . . . . . . . . . . . . . . . . . . . . . . . . . . . . . . . . . . | |
| **4** | One or two paragraphs summarizing why this case is unique (**may include** **references**) . . . . . . . . . . | . . |
| **5a** | De-identified patient specific information. . . . . . . . . . . . . . . . . . . . . . . . . . . . . . . . . . . . . . . . . . . . . . . . . . . . | |
| **5b** | Primary concerns and symptoms of the patient. . . . . . . . . . . . . . . . . . . . . . . . . . . . . . . . . . . . . . . . . . . . . . . . . . . . . | |
| **5c** | Medical, family, and psycho-social history including relevant genetic information . . . . . . . . . . . . . . . | . . |
| **5d** | Relevant past interventions with outcomes . . . . . . . . . . . . . . . . . . . . . . . . . . . . . . . . . . . . . . . . . . . . . . . . . . . . . . | . . |
| **6** | Describe significant physical examination (PE) and important clinical findings. . . . . . . . . . . . . . . . . . . . . | . . |
| **7** | Historical and current information from this episode of care organized as a timeline . . . . . . . . . . . . . . . | |
| **8a** | Diagnostic testing (such as PE, laboratory testing, imaging, surveys). . . . . . . . . . . . . . . . . . . . . . . . . . . . . | . . |
| **8b** | Diagnostic challenges (such as access to testing, financial, or cultural) . . . . . . . . . . . . . . . . . . . . . . . . . . . . . | |
| **8c** | Diagnosis (including other diagnoses considered) . . . . . . . . . . . . . . . . . . . . . . . . . . . . . . . . . . . . . . . . . . . . . . . | . . |
| **8d** | Prognosis (such as staging in oncology) where applicable . . . . . . . . . . . . . . . . . . . . . . . . . . . . . . . . . . . . . . . . . | |
| **9a** | Types of therapeutic intervention (such as pharmacologic, surgical, preventive, self-care) . . . . . . . . . . . . . . . . . . | . . |
| **9b** | Administration of therapeutic intervention (such as dosage, strength, duration) . . . . . . . . . . . . . . . . . . . . . . . . . . . . . | |
| **9c** | Changes in therapeutic intervention (with rationale) . . . . . . . . . . . . . . . . . . . . . . . . . . . . . . . . . . . . . . . . . . . . . . . . . . . . | |
| **10a** | Clinician and patient-assessed outcomes (if available) . . . . . . . . . . . . . . . . . . . . . . .. . . . . . . . . . . . . . . . . . . . . . . . . . | . . |
| **10b** | Important follow-up diagnostic and other test results . . . . . . . . . . . . . . . . . . . . . . . . . . . . . . . . . . . . . . . . . . . . . . . . . . | . . |
| **10c** | Intervention adherence and tolerability (How was this assessed?) . . . . . . . . . . . . . . . . . . . . . . . . . . . . . . . . . . . . . . . | . . |
| **10d** | Adverse and unanticipated events . . . . . . . . . . . . . . . . . . . . . . . . . . . . . . . . . . . . . . . . . . . . . . . . . . . . . . . . . . . . . . . . . | . . |
| **11a** | A scientific discussion of the strengths AND limitations associated with this case report . . . . . . . . . . . . . . . . . . . . . | . . |
| **11b** | Discussion of the relevant medical literature **with references**. . . . . . . . . . . . . . . . . . . . . . . . . . . . . . . . . . . . . . . . | . . |
| **11c** | The scientific rationale for any conclusions (including assessment of possible causes) . . . . . . . . . . . . . . . . . . . . . . . . | |
| **11d** | The primary “take-away” lessons of this case report (without references) in a one paragraph conclusion . . . . . | . . |
| **12** | The patient should share their perspective in one to two paragraphs on the treatment(s) they received . . . . . . . . | |
| **13** | Did the patient give informed consent? Please provide if requested . . . . . . . . . . . . . . . . . . . . . . . . . . . . . . . . . . . . | . . |

**Reported on Line**


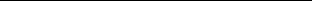

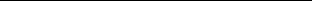

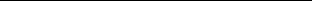

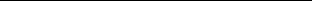

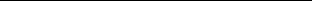

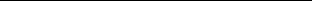

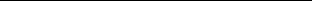

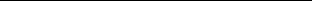

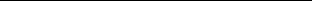

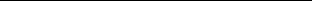

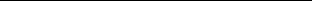

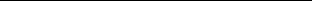

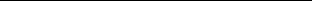

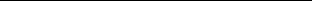

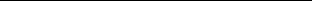

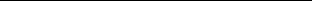

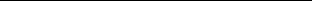

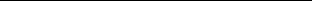

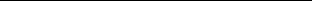

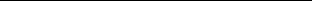

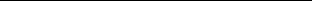

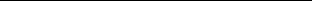

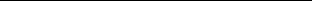

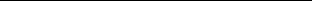

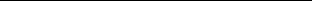

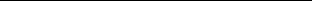

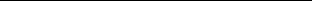

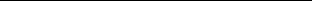

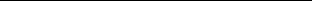


Paragraph 1/ page 1

Paragraph 2/ page 1

Paragraph 3/ page 1

Paragraph 3/ page 1

Paragraph 3/ page 1

Paragraph 1/ page 2

Paragraph2 / page 2

Paragraph 3/ page 2

Paragraph 4/ page 2

Paragraph 4/ page 2

Paragraph 4/ page 2

Paragraph 5/ page 2

Paragraph 1/ page2

Paragraph 1/ page 3

Paragraph 1/ page 3

Paragraph 2/ page 3

Paragraph 2/ page 3

Paragraph 1/ page4

Paragraph 1/ page4

Paragraph 1/ page4

Paragraph 2/ page4

Paragraph 2/ page4

Paragraph 2/ page4

Paragraph 2/ page4

Paragraph 3/ page4

Paragraph 3/ page4

Paragraph 1/ page5

Paragraph 2/page6

Paragraph 3/ page6

Yes

**Yes**
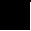

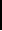
 **No**


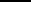

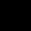

Supplement: Supplementary file 1 [file Table_1.DOCX]
